# A patient with constitutional ring 1 chromosome characterized by SNP array CGH

**DOI:** 10.1002/ccr3.522

**Published:** 2016-03-21

**Authors:** Sheila Saliganan, Joanna Lee, Sainan Wei

**Affiliations:** ^1^ Division of GeneticsDepartment of Pediatrics and Human DevelopmentCollege of Human MedicineMichigan State UniversityEast LansingMichigan; ^2^ Department of Pathology and Laboratory MedicineCollege of MedicineUniversity of Kentucky

**Keywords:** 1q43q44 deletion, array comparative genomic hybridization, chromosome 1, cytogenetics, dwarfism, growth retardation, intellectual disability, microcephaly, ring 1 chromosome, ring syndrome

## Abstract

We present a male patient with constitutional ring 1 chromosome and subsequent 6 Mb deletion at 1q43q44. The patient displays overlapping clinical features with reported patients with ring 1 chromosome and 1q43q44 microdeletion syndrome. To our knowledge, this is the first patient with ring 1 chromosome characterized by comparative genomic hybridization.

## Introduction

Constitutional ring chromosomes are a rare cytogenetic abnormality found in humans believed to form by deletion and subsequent fusion at the telomeres. In addition to deleted genomic material, the instability of the ring structure itself may contribute to an abnormal phenotype, specifically growth failure 1. It has also been suggested that larger ring structures are more labile and result in more severe growth retardation than smaller rings 2. While ring structures have been reported in various autosomes, only six patients with constitutional ring 1 chromosome have been reported to date 1, 3, 4, 5, 6, 7. The oldest reported individual lived to be at least 12 years of age 3. Consistently overlapping features in ring 1 chromosome include: microcephaly, low birth weight with severe postnatal growth retardation and dwarfism, intellectual disability, and mild dysmorphic features 4. However, the paucity of reported individuals with ring 1 chromosome limits delineation of a distinct syndrome and provides few opportunities to perform additional cytogenetic investigations.

The relatively recent advent of array comparative genomic hybridization allows for further characterization of various microdeletion syndromes. Of particular interest to patients with ring chromosome structures are telomeric deletions (1q44 and 1p36 for chromosome 1). Chromosome 1q43q44 deletion syndrome (OMIM 612337) has been reported in over 70 individuals 8 and has a recognizable phenotype, including: “intellectual disability, prenatal growth retardation, severe microcephaly, hypospadias, corpus callosum abnormalities, cardiac anomalies, gastroesophageal reflux, and characteristic facies” 9. The facial features associated with 1q43q44 deletion include: round face, flat nasal bridge, epicanthal folds, hypertelorism, and low‐set ears; however, various other features have also been reported 8, 9, 10. Several candidate genes have been identified by analyzing the smallest regions of overlap in affected individuals. For example, seizures are thought to be associated with deletion of *FAM36A, HNRNPU*, and *C10RF199* genes, agenesis of the corpus callosum due to deletions of *ZNF238* and *AKT3*, and microcephaly due to deletion of *AKT3* 8, 9, 10.

Based on the clinical overlap between patients with 1q43q44 deletion and those with ring 1 chromosome, it seems reasonable to suggest that the deletion of this region during formation of the ring structure is at least partially responsible for the abnormal phenotype. However, the characterization of deleted regions in reported patients with constitutional ring 1 chromosome has been cytogenetically limited to traditional banding techniques. Additionally, the ring structure further complicates interpretation of genotype‐phenotype correlations in these patients, as mitotic instability may contribute to a “ring syndrome” that is not entirely explained by microdeletions alone.

Here, we present a 36‐month‐old male with a constitutional ring 1 chromosome. To our knowledge, this is the first patient with a ring 1 chromosome that has been characterized by comparative genomic hybridization. His confirmed 1q43q44 deletion encompasses the *FAM36A, HNRNPU, C10RF199, ZNF238,* and *AKT3* candidate genes, among many others. He displays a number of clinical signs consistent with the previously described phenotypes of ring 1 chromosome and chromosome 1q43q44 deletion syndrome. Our patient also shows novel clinical features, which may represent incidental findings or an expanded clinical phenotype of this chromosomal abnormality. This patient report provides additional data on ring 1 chromosome and potential genotype‐phenotype correlations.

## Materials and Methods

Chromosome analyses were performed on cultured lymphocytes using G banding (GTL) techniques. Peripheral blood was collected in a sodium heparin tube. Twenty‐seven hours cultures were set up with PHA, routine culture harvesting and G banding were performed. At least 20 metaphase cells were analyzed at 550 band resolution. Fluorescent in situ hybridization (FISH) was performed using a tricolor probe set that hybridizes to band 1p36 (CEB108/T7,P58) and to band 1q25 (P58) (Vysis, Inc.) as a control. The FISH impression is based on analysis of at least 10 metaphase and 100 interphase cells.

Whole‐genome PCR amplification was performed on DNA extracted from peripheral blood or cultured cells followed by hybridization, staining, washing, and scanning by the Affymetrix CytoScan HD array. This array contains 2.67 million copy number markers/probes (1.9 million nonpolymorphic probes/markers and 750,000 SNP probes/markers) that detect copy number variations (CNVs), loss of heterozygosity (LOH), and segmental or whole chromosome uniparental isodisomy. Data is analyzed by ChAS 1.2 and compared against a reference model file provided by Affymetrix to detect gains and losses.

## Clinical Report

Our patient is a 36‐month‐old mixed Northern European, Caucasian male born to a 23‐year‐old primigravida woman. The pregnancy history was remarkable for suboptimal brain and cardiac anatomy and intrauterine growth restriction identified on 20 week ultrasound. Amniocentesis revealed the following karyotype: 46,XY,r(1)(p36.3q44)^10^/45,XY,‐1^5^ (fig. 3). Ten colonies had a ring chromosome 1 with losses of the bands of 1p36.3 and 1q44; the remaining five colonies had 45 chromosomes with a loss of chromosome 1, which possibly resulted from culture artifact or is true mosaicism – both reflecting ring chromosome instability. FISH confirmed a deletion of 1p36 on the ring chromosome in amniocytes.

Chromosome analysis in peripheral blood on DOL 1 confirmed 20 metaphase cells each with a ring 1 chromosome: 46,XY,r(1)(p36.3q44). Parental chromosome studies were normal. Chromosomal microarray of our patient revealed a copy number loss of 6744 probes, estimated to be at least 6 Mb in size, in the region of 1q43q44 (fig. 4). This confirmed the q‐arm breakpoint to be at 1q43q44. Due to the paucity of probes with this array platform at the 1p telomere, it was not possible to define the p‐arm breakpoint although it was cytogenetically assessed to be at 1p36.3. There was no loss of 1‐pter by array or FISH in peripheral blood detected.

**Figure 1:**
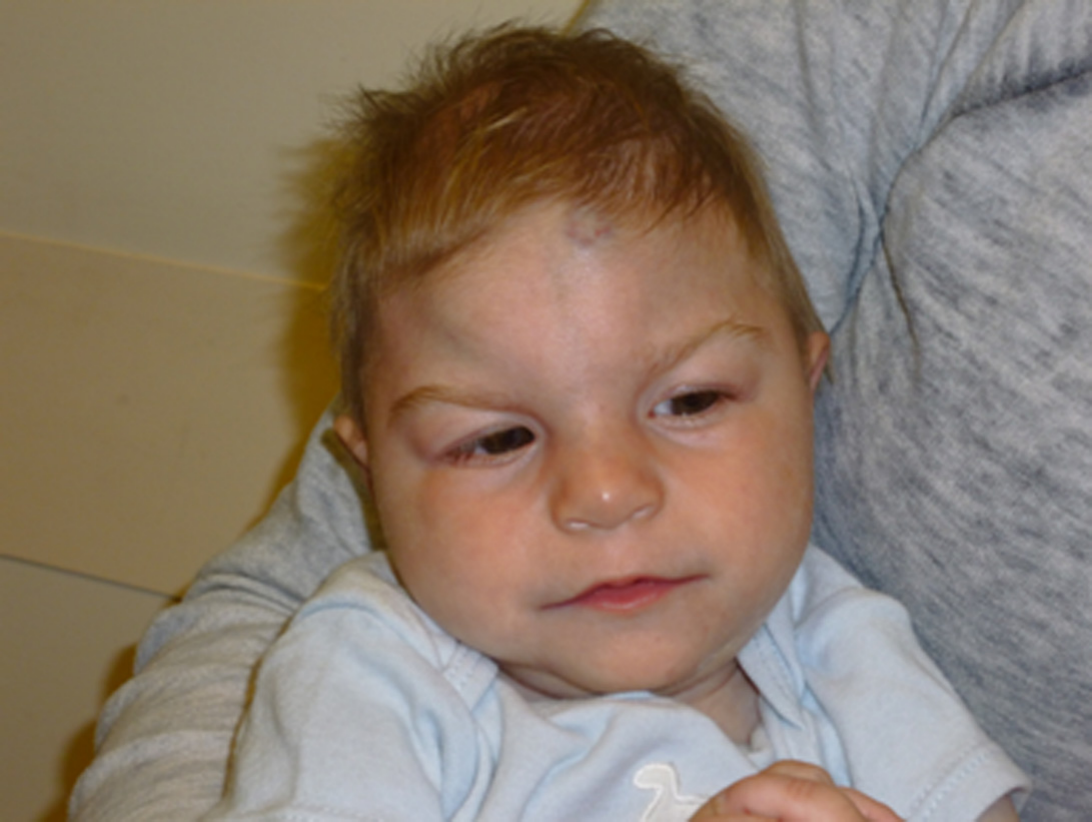
Dysmorphic facial features of patient including: thin lip, prominent nasal root, large ears, sagittal ridging, lateral flaring of the eyebrows, and glabellar hemangioma.

The patient was born at 41 weeks gestation weighing 1.90 kg (−3 SD below the mean) with a length of 40.5 cm (−4 SD below the mean), head circumference of 28 cm (−3.5 SD below the mean), and Apgar scores of 7 and 8 at 1 and 5 min, respectively. Examination at birth noted significant microcephaly and subtle dysmorphic features (fig. 1). The patient failed his newborn hearing screen bilaterally and confirmatory audiology exam revealed mild hearing loss in the left, which improved after tube placement. Newborn metabolic screen was positive for hypothyroidism, which has since been effectively managed with levothyroxine. He also had a history of neonatal thrombocytopenia, which resolved spontaneously. Ophthalmology consult in the RNICU was unremarkable. Due to a sacral pit, a spinal ultrasound was performed and was normal. A skeletal survey including AP and lateral X‐rays of bilateral upper and lower extremities was performed on DOL 2 due to the chromosomal abnormality and was negative for congenital anomalies.

Cerebral MRI without contrast performed due to microcephaly on DOL 1 revealed primitive/premature appearance to the sulci and gyral pattern. Due to a small anterior fontanelle and absent palpable posterior fontanelle, a head CT including axial images with coronal and sagittal recontructions without contrast and 3D reconstruction was performed on DOL 3 and was a negative study with no evidence for craniosynostosis. The patient receives serial head CT scans to monitor a large glabellar hemangioma, and these have also been negative for craniosynostosis. Head CT at 23 months of age identified a new lesion in the region of foramen of Monro suspicious for brain tumor; brain MRI with and without contrast at 23 months of age confirmed a 1.86 × 1.11 cm enhancing mass in the midline, occupying much of the third ventricle. Differential diagnoses include: choroid plexus papilloma or carcinoma, ependymoma, or germ cell tumor. Dysgenesis of the corpus callosum was also incidentally noted on this MRI scan. An awake/sleep EEG performed at 14 months of age due to an episode of apnea and cyanosis revealed slow waves and diffuse encephalopathy, but was negative for seizure activity. Significant developmental delays have been noted since birth. At the most recent genetics evaluation at 17 months of age, he was functioning at the level of a 4‐month‐old. At 23 months, he was functioning at the level of a 2‐ to 3‐month‐old per the family's assessment.

**Figure 2:**
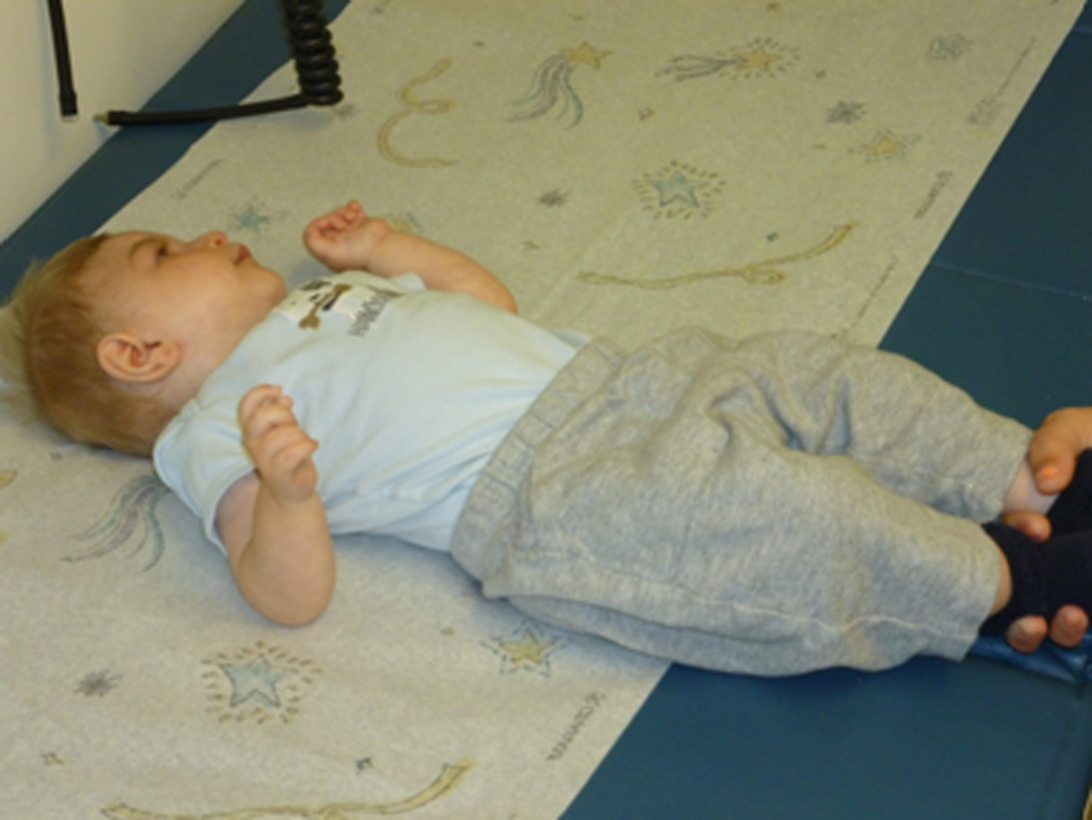
Patient at chronological age of 17 months had a height of 60.33 cm, weight of 4.52 kg, and head circumference of 33 cm, all significantly below the 5th percentile.

The patient also has a history of penile phimosis, left inguinal hernia, and unilateral cryptorchidism, all of which were successfully repaired. A small patent ductus arteriosis and atrial septal defect resolved spontaneously. His height at 17 months of age was 60.33 cm (−7.5 SD below the mean), weight was 4.52 kg (−7 SD below the mean), and head circumference was 33 cm (−12 SD below the mean) (fig. 2). At 23 months of age, height was 60.5 cm (−8 SD below the mean), weight was 4.6 kg (−7 SD below the mean), head circumference was 34 cm (−11 SD below the mean). Additional features are summarized in Table 1. The family history was remarkable for thyroid dysfunction in the patient's father, paternal aunt, two maternal aunts, and maternal grandmother. The family history was otherwise noncontributory.

**Figure 3:**
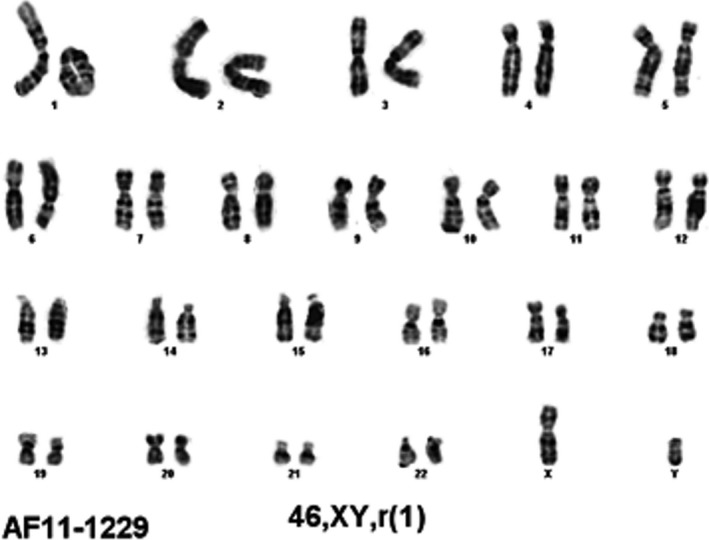
Chromosome analysis in amniocytes revealed constitutional ring 1 chromosome with breakpoints cytogenetically assessed to be at 1q44 and 1p36.3 (46,XY,r(1)(p36.3q44)).

**Figure 4:**
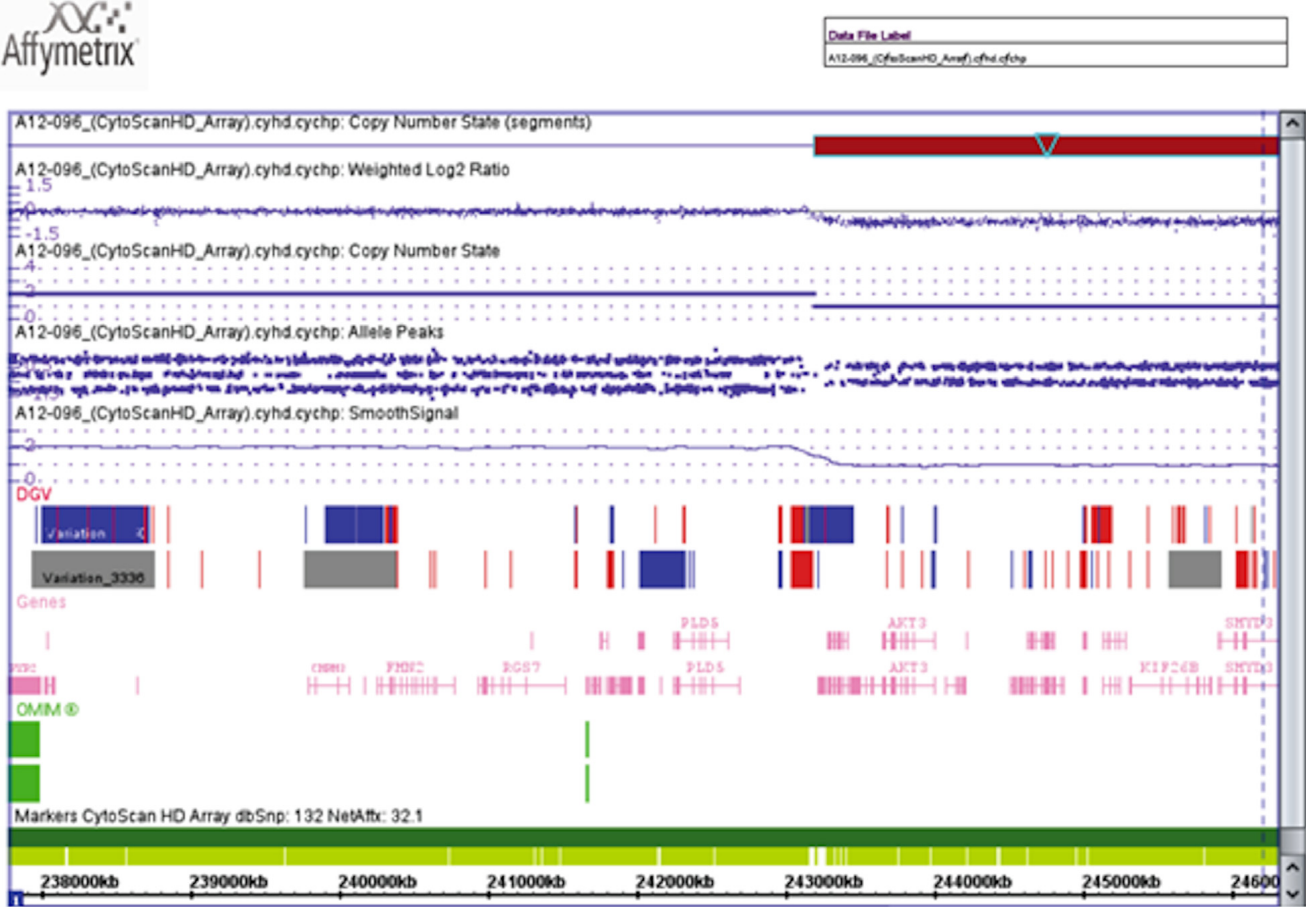
Chromosomal microarray revealed 1q43q44 (arr 1q43q44(243,204,375‐249,224,684)×1).

**Table 1:** Clinical features of patients with constitutional ring 1 chromosome syndrome

	Present case	Gordon and Cooke 6	Wolf et al. 7	Bobrow et al. 3	Kjessler et al. 1	Gardner et al. 5	Cutenese et al. 4
	46,XY, r(1)(p36.3q44)	46,XX, r(1)	46,XX, r(1)	46,XX, r(1)	46,XX, r(1)	46,XX, r(1)	46,XX, r(1)(p36.3q44)
Birth parameters	BW: 1.90 kg	BW: 1.64 kg	BW: 1.08 kg	BW: 1.80 kg	BW: 1.69 kg	BW: 1.48 kg	BW: 1.67 kg
	BL: 40.5 cm	BL: N/A	BL: N/A	BL: N/A	BL: 39 cm	BL: 42 cm	BL: 41 cm
	OFC: 28 cm	OFC: 28.6 cm	OFC: N/A	OFC: 28 cm	OFC: 26 cm	OFC: 28 cm	OFC: 29.25 cm
Growth retardation/Dwarfism	+	+	+	+	+	+	+
Microcephaly	+	+	+	+	±	+	+
ID/DD	+	+	+	+	+	+	+
Dysmorphic facies	+	+	+	+	+	+	+
	Large ears Prominent nose Thin lip Sagittal ridging Lateral eyebrow flare Glabellar hemangioma Small anterior fontanelle	Small anterior fontanelle	Large ears Lowset ears “Elfin” facies Upturned mouth	High nasal bridge Upslanting PF Pre‐auricular sinuses	Large, dysplastic ears Micrognathia Upslanting, small PF Epicanthal folds Prominent occiput Long philtrum Small nose Depressed nasal bridge Small anterior fontanelle	Prominent nose Micrognathia Thin lip Sloped forehead	Prominent nose Micrognathia Upslanting, short PF High/broad forehead Lowset ears
Cardiac involvement	+	−	−	−	+	−	+
	Atrial septal defect Patent ductus arteriosis				Heart murmur RV and RA hypertrophy		Atrial septal defect
Musculoskeletal involvement	+	−	+	+	+	+	+
	Rocker bottom feet Single palmar creases Possible lack of interosseus muscles in fingers		Hypotonia Thin extremities Decreased muscle mass Congenital absence of teeth	Clinodactyly Hip dislocation Talipes cavus Hypertonia Cerebral palsy Tapered fingers	Clinodactyly Hypotonia Hip dislocation Retroposition of 4th toe Short extremities/fingers Split vertebra	Hypoplastic thumbs Radially deviated hands Absent 1st metacarpals Abnormal tendinous attachment of thumbs	Rocker bottom feet Clinodactyly Single flexion crease
Genitourinary involvement	+	−	−	−	−	−	+
	Cryptorchidism Inguinal hernia Penile phimosis						Dysplasic right kidney Renal cyst Vesicoureteric reflux
Other functional abnormalities	**+**	−	**+**	**+**	**+**	−	−
	Mild hearing loss Hypothyroidism Transient thrombocytopenia Diffuse encephalopathy		Abnormal growth hormone	Anemia Acute myeloid leukemia	Ascites Hepatosplenomegaly Vomiting daily Tube fed		
Other structural abnormalities	+	−	−	−	+	−	+
	High arched palate Anterior placed anus Sacral pit Primitive sulci/gyral pattern DCC Brain tumor				Macrocephaly Hydrocephalus Cleft palate		DCC

BL, birth length; BW, birth weight; OFC, occipitofrontal head circumference; PF, palpebral fissures; RA, right atrium; RV, right ventricle; ID, intellectual disability; DD, developmental delay; DCC, dysgenesis of corpus callosum.

## Discussion

We present the first patient with pure, constitutional ring 1 chromosome to be characterized by SNP CGH microarray. A review of six previously reported patients with ring 1 chromosome reveals several overlapping clinical features with the current patient (Table 1). All reported patients with a ring 1 chromosome, including ours, had significant growth retardation, intellectual disability, and facial dysmorphisms, and all but one had microcephaly (this individual instead had macrocephaly due to hydrocephalus). Clinical features that are reported in two or more of the seven reported patients with ring 1 chromosome include: large ears, low‐set ears, prominent nose, micrognathia, thin lip, small PF, upslanted PF, small anterior fontanelle, dysgenesis of corpus callosum, atrial septal defect, rocker bottom feet, clinodactyly, hypotonia, single palmar crease, and congenital hip dislocation. The remaining features are isolated to one report (see Table 1). This data should be interpreted with caution due to the very small number of patients reported, which precludes any accurate delineation of a syndrome. Additionally, characterization of deleted regions in patients with constitutional ring 1 chromosome has previously been limited to traditional cytogenetic techniques.

Conversely, 1q43q44 microdeletion is better characterized with over 70 reported patients 8. Several features reported in individuals with ring 1 chromosome overlap with those carrying this microdeletion, including: microcephaly, poor growth, developmental delay or intellectual disability, dysgenesis of the corpus callosum, minor cardiovascular malformations (i.e., atrial septal defect), limb anomalies (i.e., clinodactyly, hip dislocation), genitourinary anomalies (i.e., inguinal hernia), and some facial dysmorphisms (i.e., low‐set ears). However, there are features, such as seizures, that have been reported in patients with 1q43q44 deletion but not ring 1 chromosome, and the growth retardation appears to be more significant in individuals with ring 1 chromosome than those with 1q43q44 deletion.

Our patient is the first with constitutional ring 1 chromosome to be characterized by SNP array CGH to our knowledge. The patient presented here has a pure ring 1 chromosome that resulted in a deletion at 1q43q44, confirmed by SNP array CGH. Thus, he presents with clinical features that overlap both with reported patients with ring 1 chromosome and those with 1q43q44 microdeletion syndrome. Features that are unique to our patient are cryptorchidism, brain tumor and primitive sulci/gyral pattern on brain MRI, slow waves and diffuse encephalopathy on EEG, mild hearing loss, congenital hypothyroidism, transient neonatal thrombocytopenia, glabellar hemangioma, and pedal edema. This report contributes towards a better understanding of clinical features and potential genotype‐phenotype correlations in patients with constitutional ring 1 chromosome.

There is a long‐standing debate as to whether the phenotype in individuals with ring chromosomes is attributed to a “ring syndrome” due to mitotic instability or to the loss of specific genes due to formation of the ring chromosome 1, 2. Because dwarfism is reported in individuals with ring 1 chromosome (and other ring syndromes), but not in patients with 1q43q44 deletion, we suspect that our patient's significant growth retardation is the result of the former, as this would not be entirely explained by his 1q43q44 deletion. However, we were unable clarify the breakpoints on the p‐arm of chromosome 1 for our patient and therefore cannot rule out a p‐arm terminal deletion that may be contributing to his growth retardation. Additionally, our comparisons are limited by the small number of patients reported with a constitutional ring 1 chromosome and 1q44q43 deletion syndrome, likely with varying breakpoints and genes involved. Therefore, more studies are needed to further delineate the nature of ring 1 chromosome syndrome. This patient report provides additional data on ring 1 chromosome and potential genotype‐phenotype correlations.

## Conflict of Interest

The authors declare no conflicts of interest.
